# Validation of a theoretically based mental health literacy framework: A meta-analytic structural equation modeling approach

**DOI:** 10.1192/j.eurpsy.2023.1027

**Published:** 2023-07-19

**Authors:** Y.-J. Lien, L. Chen

**Affiliations:** National Taiwan Normal University, Taipei, Taiwan, Province of China

## Abstract

**Introduction:**

Mental Health Literacy (MHL) includes four distinct but interrelated components: maintenance of positive mental health (**MH**), recognition of mental disorders (**R**), mental illness stigma attitudes (**S**), and help-seeking efficacy (**E**). A fifth component, help-seeking attitude (**A**) was included in a MHL model because it is a strong predictor of help-seeking behaviors. The five-factor model of MHL has been validated previously. In the study of multiple mediation model of MHL, it demonstrated the mediation role of help-seeking efficacy between recognition of mental disorders and help-seeking attitude (**R-E-A** path), but the roles of mental illness stigma attitudes or maintenance of positive mental health remain unknown.

**Objectives:**

The present study aimed to examine the theory-based multifaceted MHL model by utilizing a meta-analytic structural equation modeling analyses (MASEM) method.

**Methods:**

Systematic search of articles from electronic databases, including APA PsycArticles, ERIC, MEDLINE, Psychology and Behavioral Sciences Collection, Pubmed, and Airiti Library (Chinese), from inception up to July 31, 2022, was conducted by raters independently assessed study eligibility, and extracted 127 empirical, quantitative, non-interventional studies with properly reported effect sizes (k=192). MASEM analyses were conducted via a two-stage approach. First, a pooled correlation matrix was obtained for each mediation model by applying a multivariate random-effects model using Comprehensive Meta-Analysis Version 3.3. Second, a structural equation model was fitted on the pooled correlation matrices to test for mediation effects (i.e., indirect effects) using IBM SPSS Amos.

**Results:**

In the single-mediator model analysis, there was a significant partial mediation effect of help-seeking efficacy and Mental illness stigma attitudes on the relationship between recognition of mental disorders and help-seeking attitude (R-E-A path & R-S-A path, p <.05). The multiple-mediator model has showed adequate fit (RMSEA = 0.09, SRMR = 0.04, CFI = 0.93, GFI = .99). It confirmed the help-seeking efficacy served as a mediator. A sequential mediation of maintenance of positive mental health and mental illness stigma attitudes was found (p <.05) between recognition of mental disorders and help-seeking attitude (R-MH-S-A path).

**Conclusions:**

There is a robust mediation effect of Help-seeking efficacy on the relationship between recognition of mental disorders and help-seeking attitude either in the single mediation model or the multiple mediation model. Furthermore, increased recognition of mental disorder was related to increased maintenance of positive mental health, therefore decreased mental illness stigma attitude, and eventually increased help-seeking attitude. Future research directions regarding pathways in the MHL models were discussed.

**Disclosure of Interest:**

None Declared

